# Labile Heme Aggravates Renal Inflammation and Complement Activation After Ischemia Reperfusion Injury

**DOI:** 10.3389/fimmu.2019.02975

**Published:** 2019-12-20

**Authors:** Li Wang, Vijith Vijayan, Mi-Sun Jang, Anja Thorenz, Robert Greite, Song Rong, Rongjun Chen, Nelli Shushakova, Igor Tudorache, Katja Derlin, Pooja Pradhan, Kukuh Madyaningrana, Nodir Madrahimov, Jan Hinrich Bräsen, Ralf Lichtinghagen, Cees van Kooten, Markus Huber-Lang, Hermann Haller, Stephan Immenschuh, Faikah Gueler

**Affiliations:** ^1^Department of Nephrology, Hannover Medical School, Hanover, Germany; ^2^Institute for Transfusion Medicine, Hannover Medical School, Hanover, Germany; ^3^Department of Cardiothoracic Surgery, Hannover Medical School, Hanover, Germany; ^4^Institute for Diagnostic and Interventional Radiology, Hannover Medical School, Hanover, Germany; ^5^Department of Pathology, Hannover Medical School, Hanover, Germany; ^6^Department of Laboratory Medicine, Hannover Medical School, Hanover, Germany; ^7^Department of Nephrology, Leiden University Medical Centre, Leiden, Netherlands; ^8^Institute of Clinical and Experimental Trauma-Immunology, University Hospital of Ulm, Ulm, Germany

**Keywords:** ischemia reperfusion injury, AKI, HO-1, C5aR, C3aR, complement

## Abstract

**Background:** Ischemia reperfusion injury (IRI) plays a major role in solid organ transplantation. The length of warm ischemia time is critical for the extent of tissue damage in renal IRI. In this experimental study we hypothesized that local release of labile heme in renal tissue is triggered by the duration of warm ischemia (15 vs. 45 min IRI) and mediates complement activation, cytokine release, and inflammation.

**Methods:** To induce IRI, renal pedicle clamping was performed in male C57BL/6 mice for short (15 min) or prolonged (45 min) time periods. Two and 24 h after experimental ischemia tissue injury labile heme levels in the kidney were determined with an apo-horseradish peroxidase assay. Moreover, renal injury, cytokines, and C5a and C3a receptor (C5aR, C3aR) expression were determined by histology, immunohistochemistry and qPCR, respectively. In addition, *in vitro* studies stimulating bone marrow-derived macrophages with LPS and the combination of LPS and heme were performed and cytokine expression was measured.

**Results:** Inflammation and local tissue injury correlated with the duration of warm ischemia time. Labile heme concentrations in renal tissue were significantly higher after prolonged (45 min) as compared to short (15 min) IRI. Notably, expression of the inducible heme-degrading enzyme heme oxygenase-1 (HO-1) was up-regulated in kidneys after prolonged, but not after short IRI. C5aR, the pro-inflammatory cytokines IL-6 and TNF-α as well as pERK were up-regulated after prolonged, but not after short ischemia times. Consecutively, neutrophil infiltration and up-regulation of pro-fibrotic cytokines such as CTGF and PAI were more pronounced in prolonged IRI in comparison to short IRI. *In vitro* stimulation of macrophages with LPS revealed that IL-6 expression was enhanced in the presence of heme. Finally, administration of the heme scavenger human serum albumin (HSA) reduced the expression of pro-inflammatory cytokines, C3a receptor and improved tubular function indicated by enhanced alpha 1 microglobulin (A1M) absorption after IRI.

**Conclusions:** Our data show that prolonged duration of warm ischemia time increased labile heme levels in the kidney, which correlates with IRI-dependent inflammation and up-regulation of anaphylatoxin receptor expression.

## Introduction

Ischemia reperfusion injury (IRI) is a major complication in solid organ transplantation (1). Cold ischemia time (CIT) for kidney allografts after post mortal donation can reach >20 h which mediates delayed graft function (DGF). In contrast, living donation has only 2–3 h CIT and lower rates of about 5% DGF (2). Notably, prolonged warm ischemia times have been linked to DGF observed in obese recipients who have undergone complicated surgeries (3). Additionally, IRI-mediated acute kidney injury (AKI) is a frequently encountered complication in other forms of solid organ transplantation such as lung transplantation (4).

The kidney is very sensitive to hemolysis-mediated damage (5). Hemolysis- associated hemoglobin cast nephropathy has been reported in renal biopsies of patients with various conditions such as autoimmune hemolytic anemia, paroxysmal nocturnal hemoglobinuria, transfusion of incompatible blood, disseminated intravascular coagulation (DIC) and in hemoglobinopathies (6). Hemolytic uremic syndrome (HUS) also leads to chronic kidney disease (CKD) and even end stage renal disease (ESRD) (7). Enhanced nephrotoxicity has also been documented in an experimental model of trauma hemorrhage (8). Upon hemolysis extracellular hemoglobin gets oxidized to methemoglobin and releases its prosthetic heme group. This fraction of “free or loosely” bound heme also termed “labile” is biologically active and high levels of labile heme are considered to be cytotoxic and to aggravate inflammation and tissue injury (9, 10). In support of this notion are the findings that the deficiency of the heme-degrading enzyme heme oxygenase-1 aggravates renal injury in different models of nephrotoxicity (11, 12).

In the current study we hypothesized that labile heme contributes to IRI-induced kidney injury. To test this hypothesis we determined labile heme levels in the kidney in a mouse model of renal IRI. Further, we also investigated the correlation between IRI-induced labile heme levels, inflammation and AKI. A panel of early and late inflammatory and histopathological markers after short (15 min) and prolonged (45 min) warm ischemia times were determined. In addition, the effect of administration of human serum albumin (HSA) as a heme scavenger was investigated in the 45 min IRI model.

## Materials and Methods

### Animals

All experiments were performed with adult male C57Bl/6 mice (11–13 weeks of age, bodyweight 23–28 g). Mice were housed and bred in the Institute of Laboratory Animal Sciences, Hannover Medical School. Mice had free access to drinking water and food. The day/night cycle was 14/10 h. Mice were monitored daily for the physical condition after surgery. The experiments were approved by the local animal protection committee of the Lower Saxony State department for animal welfare and food protection (33.19-42502-04-14/1657) which are in line with the National Institutes of Health guidelines.

### Renal Ischemia Reperfusion Injury

Mice were anesthetized with isoflurane (3% induction, 1–2% maintenance), for analgesic treatment butorphanol 1 mg/kg bodyweight was given subcutaneously. IRI was induced by unilateral renal pedicle clamping with a micro aneurysm clip (Aesculap, Germany) for either 15 or 45 min. Afterwards, reperfusion was visually controlled. The contralateral not clipped kidney and sham surgery with opening of the abdominal cavity served as controls. For inhibitor experiments human albumin (Kendrion Biopharma, purity 98%), was diluted by sterile Phosphate Buffered Saline (PBS) to a final dose of 4 mg /mouse given i.v. 10 min before IRI 45 min surgery. The vehicle group received PBS injection.

### Organ Preservation

Mice were sacrificed at two and 24 h after IRI and organ retrieval was done in deep general anesthesia (5% isoflurane). After midline laparotomy whole body perfusion with ice-cold 0.9% PBS via the left cannulated ventricle resulted in a circulatory arrest. Organs were dissected and fixed in RNA later, 4% paraformaldehyde or shock frozen in liquid nitrogen.

### Renal Morphology and Immunohistochemistry

After paraffin embedding 2 μm sections were cut and stained. For overall morphology Perjodic Acid Schiffs (PAS) stain was done. Semi-quantitative scoring for signs of AKI was done: 0 = none or focal AKI, <5% of tubuli affected. 1 = mild AKI, 5–25% of tubuli affected. 2 = moderate AKI, 26–50% of tubuli affected. 3 = severe AKI, 51–75% of the tubuli affected. 4 = very severe AKI > 75% of tubuli affected. Immunohistochemistry was done with the following antibodies: Gr-1^+^ for neutrophils (Ly-6G/Ly-6C^+^, Serotec, UK), Alpha 1 Microglobulin (A1M, gift from Dr. Grams Lund University, Sweden) which is a marker of tubular transport and heme oxygenase-1 (HO-1, Enzo life sciences, Switzerland). Neutrophil infiltration was scored semi-quantitatively. 0: <5 cells/view field (VF), 1: 5–10 cells/VF, 2: 11–20 cells/VF, 3: 21–50 cells/VF and 4: >50 cells/VF. HO-1 and A1M were semi-quantitatively expressed in percentage of the affected tubuli in 10 different areas. Images were captured with the same magnification. Five to six mice per group were used for all experiments. Analysis was conducted using a Leica imaging microscope at 200-fold magnification. Investigators were blinded to the animal group assignment.

### Pro-inflammatory Cytokine Expression

For cytokine analysis total mRNA was isolated using RNeasy Mini Kit (Qiagen, Hilden, Germany). Then, cDNA was synthetized with Prime Script Reverse Transcriptase reagent (Takara, Japan) from DNase-treated total RNA. qPCR was conducted on a LightCycler 96 (Roche, Penzberg, Germany) using sybrgreen primers. The following primers were used: Interleukin-6 (IL-6, Qiagen, #QT00098875), TNFα (Qiagen, #QT00104006), Monocyte Chemoattractant Protein-1 (MCP-1, Qiagen, #QT00167832), Plasminogen-Aktivator-Inhibitor Type 1 (PAI 1, Bio Tez Berlin-Buch GmbH, fwd: 5′-ATGTTTAGTGCAACCCTGGC-3′, rev: 5′-CTGCTCTTGGTCGGAAAGAC-3′), Connective tissue growth factor (CTGF, Qiagen, #QT00096131) and HO-1 (Qiagen, #QT00159915). Complement 5a receptor 1 (C5aR1, accessionnumber: NM_007577, fwd: 5′CAGGTGACCGGGGTGATGATAGC3′rev: 5′GTAGGCCAGGGACACGCACAGG3′ and Complement 5a receptor 2 (C5aR2, accession number: NM_001173550, fwd: 5′GCTGCATACAGCACAAGCA3′, rev: 5′ACCACCACCGAGTATTATGACT3′). Complement 3a receptor (C3aR, accession number: NM_009779, fwd: 5′-GTG CTT GAC TGA GCC ATG GAG T-3′, rev: 5′-CAG CAC CAG CCC ATT GCC TA-3′), Hypoxanthine phosphoribosyl transferase (HPRT, Qiagen, #QT00166768) was used as house keeper for normalization. Five to six mice per group were analyzed.

### Protein Isolation and Western Blotting

Frozen tissue samples were lysed in RIPA lysis buffer on ice, and protein was isolated. Protein quantification was done by BCA assay (Thermo Scientific, USA). Fifty micrograms of protein was loaded on a 10% polyacrylamide gel for electrophoresis. Proteins were blotted to polyvinylidene fluoride membranes (Millipore, Darmstadt, Germany). After blocking in 3% bovine serum albumin (BSA), incubation with primary antibodies was done overnight at 4°C. The following antibodies were used: pERK (44/42 kDa, 9102S), ERK1/2 (44/42 kDa, 9106S, both from Cell Signaling Technologies, USA). 14-3-3 served as loading control (28 kDa, K-19, Santa Cruz Biotechnology, USA). After washing in PBS horseradish peroxidase (HRP) conjugated secondary antibodies (Dianova, Hamburg, Germany) were incubated for 1 h at room temperature. Proteins were visualized by incubation with SuperSignal™ West Pico Chemiluminescent Substrate (Thermo Fisher Scientific, USA). To enhance signal intensity SuperSignal™ West Femto Maximum Sensitivity Substrate (Thermo Fisher Scientific, USA) was used according to the manufacturer's instructions. Bands were immediately captured by VersaDoc MP 400 System (Bio Rad, USA). Five mice per group were analyzed.

### Determination of Labile Heme With an Apo-Horseradish Peroxidase (Apo-HRP) Assay

Labile heme assay was performed in 96 well plates as previously described (13, 14) with minor adaptations for measurement in tissue samples. Immediately after sacrifice the kidney tissue samples were weighed and minced in 1 ml Hank's Balanced Salt solution (HBSS). The supernatant was centrifuged at 1,500 rpm for 5 min to remove residual tissue aggregates and transferred to a fresh tube and stored at −80°C or directly applied for the assay. Briefly, 5–20 μl of the supernatant was added in a final volume of 100 μl HBSS reaction mixture containing 0.75 μM apo-HRP (BBI Solutions, Gwent, UK) and incubated for 10 min at 4°C. In parallel hemin standards (0.25–2.5 nM) in a final volume of 100 μl reaction mixture (HBSS + apo-HRP) prepared from a stock solution of 25 nM hemin (Frontier Scientific, Logan, UT, USA) were also incubated. Then, 5 μl of samples and standards were transferred to a new 96 well plate and the assay was initiated by adding 200 μl of TMB substrate. The absorbance at 652 nm was kinetically read for 2–3 min and the time point at which the highest hemin standard gave an absorbance from 1.6 to 2 was chosen for determining the concentration of labile heme in samples. The calculated concentrations of heme were normalized with the tissue weight and expressed as pmol/mg tissue wet weight. Five mice per group were analyzed.

### *In vitro* Studies With Mouse Bone Marrow Derived Macrophages (BMDM)

Mouse bone marrow cells were differentiated into macrophages using r-MCSF as described previously (15). After 7 days of differentiation macrophages were seeded at a density of 5 × 10^5^ cells in each well of a 6-well plate and allowed to rest overnight. Stimulation with LPS (1 μg/ml) was performed in the presence or absence of hemin at the indicated doses in medium containing 1% serum for 16 h. The cells were lysed and processed for RNA isolation.

### Statistical Analysis

For statistical analysis GraphPad prism software (GraphPad Software Inc. 5.0, San Diego, CA) was used. Differences between groups were determined by one way ANOVA or student's *t*-test if two groups were compared. Data are shown as mean ± standard error (SEM). Significant differences were defined as ^*^p < 0.05, ^**^p < 0.01, ^***^p < 0.001.

## Results

### Increased Levels of Labile Heme After Prolonged Renal Ischemia Times—Correlation With HO-1 Expression

**Figure 1:**
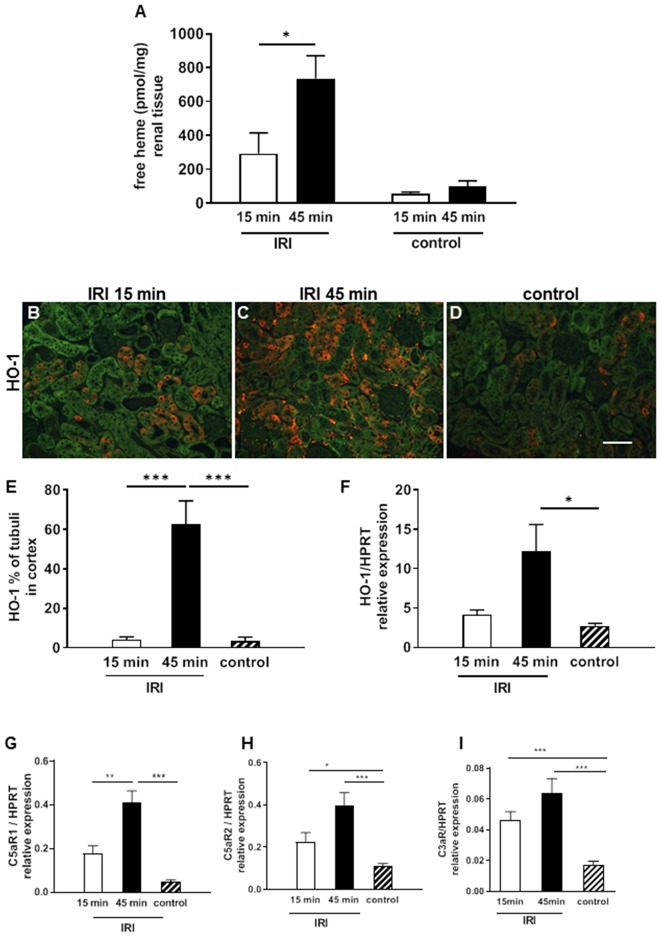
Labile heme release and complement activation after IRI. In renal tissue labile heme was elevated after 2 h in the 15 min but even more in the 45 min IRI model **(A)**. HO-1 mRNA expression increased significantly after 45 min IRI **(F)** and also the expression of HO-1 protein on proximal tubular epithelial cells was significantly enhanced in the 45 min IRI group (**B–E**, bar: 100 μm). The anaphylatoxin receptor C5aR1mRNA expression was significantly higher after prolonged ischemia time at 24 h after IRI **(G)**. C5aR2 and C3aR showed enhanced mRNA expression after 15 and 45 min IRI compared to controls but the IRI groups did not differ **(H,I)** *n* = 5 mice/group, one way ANOVA. *p < 0.05, **p < 0.01, ***p < 0.001.

IRI is worsened by the length of the warm ischemia time (16, 17). To investigate whether labile heme levels are detectable after renal IRI we used an apo-HRP based assay on renal tissue samples subjected to short (15 min) and long (45 min) time periods of warm IRI. Labile heme levels were significantly increased in IRI kidneys with prolonged (45 min) compared to 15 min IRI (Figure 1A, ^*^p < 0.05). In contrast, labile heme levels in kidney samples after sham surgery or in contralateral non-clipped kidneys which served as controls were markedly lower. To investigate whether the determined levels of labile heme in kidneys after IRI are biologically relevant, we also determined renal expression of HO-1, which is the inducible isoform of the heme-degrading enzyme HO and is highly up-regulated by heme (18, 19). Interestingly, HO-1 protein was induced in proximal renal tubuli after prolonged, but not after short ischemia times (Figures 1B–E, ^***^p < 0.001). Similarly, HO-1 mRNA expression was significantly up-regulated in 45 min IRI (Figure 1F, ^*^p < 0.05).

In addition, the mRNA expression of anaphylotoxin receptors C5aR1, C5aR2, and C3aR were analyzed as markers of complement activation. The expression of all three receptors was significantly induced after IRI (Figures 1G–I, ^***^p < 0.001). Of note, only for C5aR1 expression the difference between 15 min and 45 min IRI reached statistical significance (Figure 1G, ^**^p < 0.05).Taken together, the data show that increased levels of labile heme after prolonged warm ischemia time correlates with up-regulation of the heme-inducible gene HO-1 and the anaphylatoxin receptors in IRI kidneys.

### Regulation of Inflammation Markers After Short and Prolonged Ischemia Times in Renal IRI

**Figure 2:**
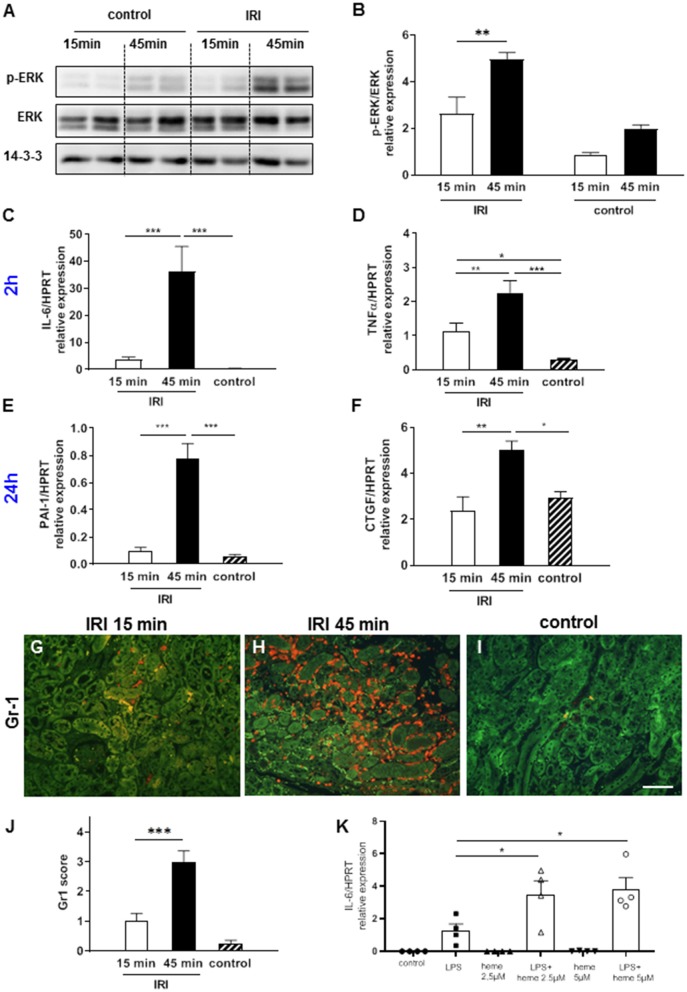
ERK activation, cytokine expression and neutrophil infiltration after IRI. Already at 2 h after IRI ERK activation was significantly enhanced in the kidneys after 45 min compared to 15 min IRI (**A,B**, *p < 0.05). Pro-inflammatory cytokines IL-6, TNF-α at 2 h **(C,D)** after IRI and pro-fibrotic cytokines PAI-1 and CTGF at 24 h **(E,F)** after IRI were significantly increased in 45 min compared to 15 min IRI. Neutrophil infiltration was mainly observed in the outer medulla and was more prominent after prolonged ischemia time at 24 h after IRI (**G–J**, bar: 100 μm, mean ± SEM. *p < 0.05, **p < 0.01, ***p < 0.001, *n* = 6 mice/group, one way ANOVA). Co-stimulation experiments with bone marrow-derived mouse macrophages revealed that heme amplified the LPS induced up-regulation of IL-6 mRNA (**K**, *p < 0.05 LPS vs. LPS+heme, one way ANOVA).

Heme has previously been shown to cause activation of the MAP kinase ERK1/2 (20) and TLR4-mediated up-regulation of pro-inflammatory cytokines TNF-α and IL-6 (21, 22). Hence, we hypothesized that an increase in labile heme after prolonged IRI will also lead to ERK1/2 activation and up-regulation of TNF-α and IL-6. Accordingly, prolonged IRI but not short IRI caused significant up-regulation of pERK at the 2 h time point (Figures 2A,B, ^**^p < 0.01). Similarly, at this time-point the expression of the pro-inflammatory cytokines IL-6 and TNF-α were also markedly up-regulated after prolonged IRI in comparison to short IRI (Figures 2C,D, ^**^p < 0.01, ^***^p < 0.001). At 24 h expression of pro-fibrotic cytokines were also altered: PAI-1 and CTGF showed significant differences between short and prolonged IRI (Figures 2E,F). We also determined renal invasion of neutrophils as a marker of inflammation. Infiltration of neutrophils was most prominent in the outer medulla and Gr1 scores were 3-fold higher after 15 min IRI and 10-fold higher in 45 min IRI kidneys compared to controls at 24 h after IRI (Figures 2G–J, ^***^p < 0.001). IRI causes activation of the TLR4 signaling pathway (23) and heme might play a role in amplifying TLR4-mediated inflammatory response. To test if heme can amplify TLR-4 mediated responses we stimulated bone marrow-derived macrophages (BMDM) *in vitro* with the TLR4-agonist lipopolysaccharide (LPS) in the presence or absence of heme. The LPS-induced up-regulation of IL-6 markedly increased in a dose-dependent manner in the presence of heme (Figure 2K, heme at 2.5 and at 5 μM, ^*^p < 0.05). To summarize, these results indicate that increased labile heme correlates with the increase in inflammatory parameters after prolonged IRI.

### Acute Kidney Injury After Short and Prolonged IRI

**Figure 3:**
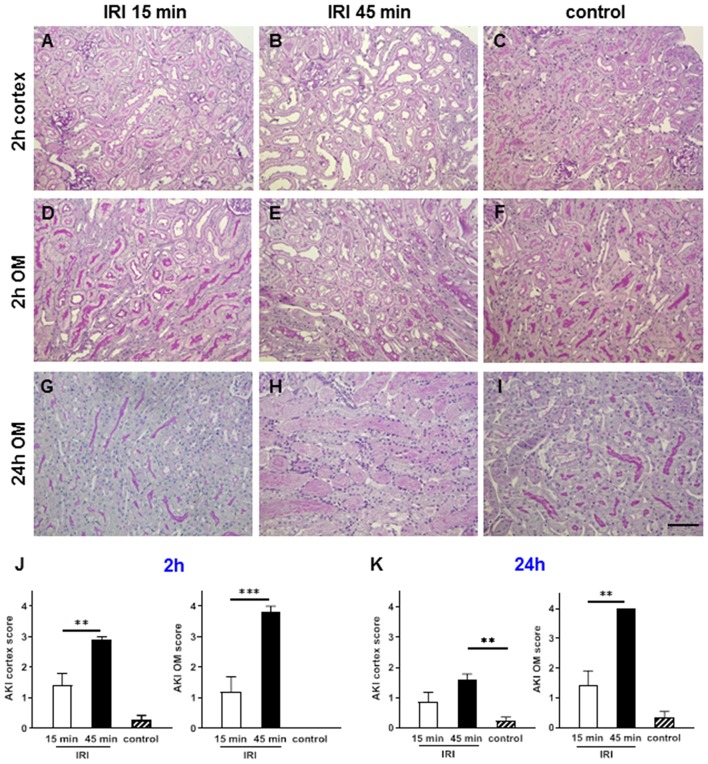
Renal morphology at two and 24 h after IRI. PAS staining revealed that mild AKI was present already after 2 h in short term IRI (15 min, **A,D,G,J**). Longer ischemia time of 45 min caused more severe AKI mainly in the outer medulla **(B,E,H,K)**. Sham kidneys served as controls and showed normal renal morphology **(C,F,I)**. Signs of AKI were less at 24 h after IRI in this short IRI group and more pronounced in the prolonged IRI group (**G–I**, mean ± SEM, **p < 0.01, ***p < 0.001, bar: 100 μm, *n* = 6 mice/group, one way ANOVA).

At 2 h after short IRI some signs of mild AKI with partial loss of the brush border membrane and tubular dilatation were detected (Figures 3A,D). As expected, prolonged IRI resulted in more severe AKI with flattening of the tubular epithelial cells, tubular cell detachment and cast formation. Major tissue damage was observed in the outer medulla where hypoxia is most severe due to the physiological lower oxygen saturation compared to the cortex (24). At 24 h after prolonged IRI the outer medulla had very severe AKI with tubular casts and interstitial inflammation, which was hardly detectable after short term IRI (Figures 3G–K).

### Impairment of Tubular Function After Prolonged IRI

**Figure 4:**
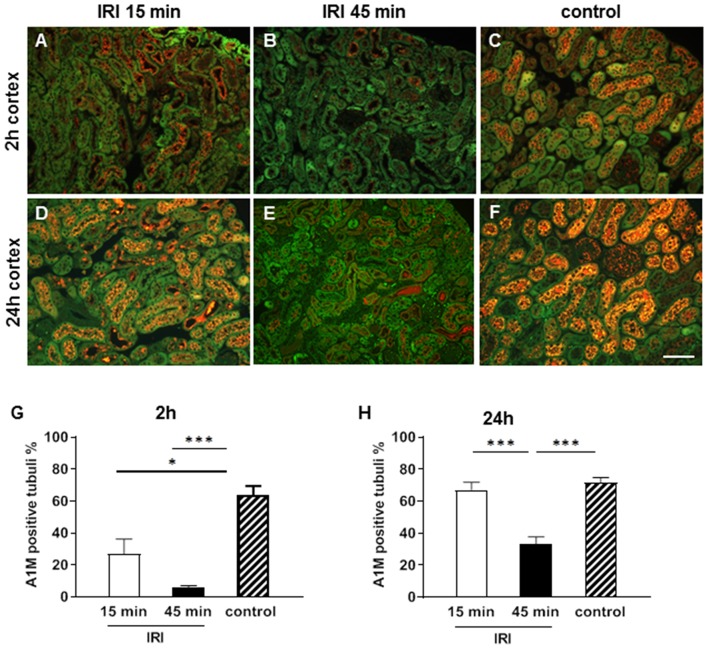
Tubular function at two **(A–C,G)** and 24 h **(D–F,H)** after IRI. A1M is reabsorbed from the intact tubuli and is stained in small vesicles in the cytoplasm of 70% of the proximal tubuli in the cortex in the healthy state **(C,F)**. Already after 2 h after short IRI A1M expression was moderately reduced **(A)** and after 45 min IRI normal vesicular A1M staining was almost absent in the pTECs **(B)**. At 24 h vesicular A1M staining was normal again in the short term IRI group **(D)** and significantly reduced in the prolonged IRI group (**E**, bar: 100 μm, mean ± SEM, *p < 0.05, ***p < 0.001, *n* = 6 mice/group, one way ANOVA).

A1M is a circulating protein that is synthesized in the liver, filtered in the glomeruli and reabsorbed by healthy proximal tubular epithelial cells (pTEC). A1M was located in small vesicles in the cytoplasma of 60% of the healthy pTECs in the control groups. Already at 2 h after IRI tubular reabsorption was disturbed and A1M expression was reduced after short and virtually abolished in prolonged IRI (Figures 4A–C,G). The reduction of tubular reabsorption was even more pronounced and almost absent after 24 h in the prolonged IRI model. The profound tubular damage caused protein cast formation in the outer medulla which also contained A1M after prolonged IRI (Figure 4E).

### Treatment With the Heme Scavenger Human Serum Albumin Attenuated IRI

**Figure 5:**
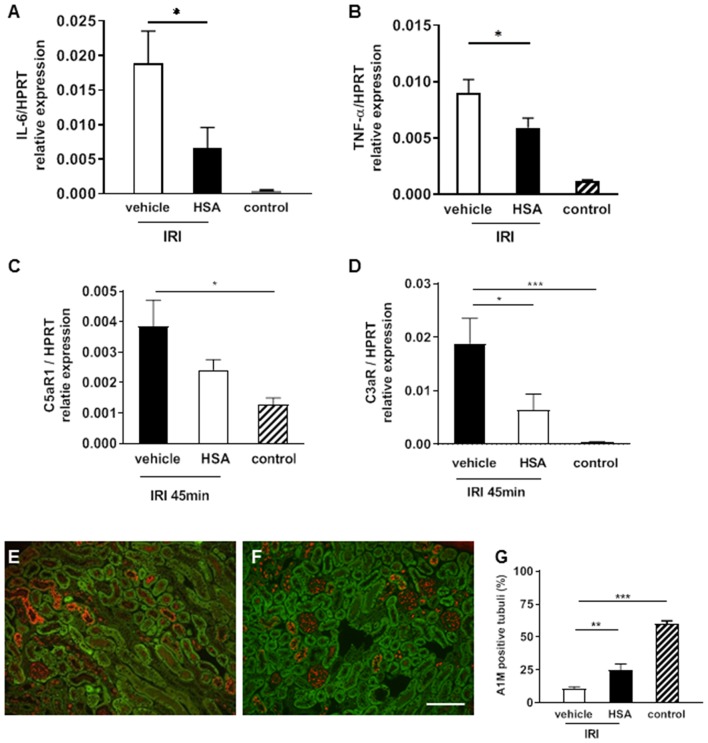
Albumin treatment to attenuate IRI. Human serum albumin (HSA) was given intravenously 10 min prior to IRI (45 min). The pro-inflammatory cytokine IL-6 and TNF-α mRNA expression was significantly decreased in the albumin treatment group compared to the vehicle at 2 h after IRI (**A,B** *p < 0.05). C5aR1 expression was significantly higher in vehicle treated IRI kidneys compared to control kidneys (**C**, *p < 0.05). C3aR mRNA expression was significantly reduced by albumin treatment compared to vehicle (**D**, *p < 0.05). The tubular function marker A1M showed higher expression after albumin treatment in proximal tubular epithelial cells after IRI (**E–G**, bar: 100 μm mean ± SEM, *p < 0.05, **p < 0.01, ***p < 0.001, *n* = 6 mice/group, one way ANOVA). *n* = 5 mice/group, one way ANOVA.

Human serum albumin (HSA), which is routinely applied in the clinic for various medical indications, is known to bind heme with high affinity and can therefore be considered as a potent heme scavenger. To investigate whether HSA may interfere with the putative heme-mediated effects of renal IRI, HSA was administered intravenously 10 min prior to prolonged (45 min) IRI. Treatment with HSA blocked the expression of pro-inflammatory cytokines IL-6 and TNF-α (Figures 5A,B, ^*^p < 0.05). Similarly, the expression of the anaphylatoxin receptor C3aR was significantly reduced by albumin treatment (Figures 5C,D, ^*^p < 0.05). In addition, A1M staining was increased in the HSA treated IRI group (Figures 5E–G) indicating improved maintenance of tubular function by absorption of A1M. To summarize, these results indicate that treatment with albumin may at least partially prevent IRI-mediated inflammatory activation and damage.

## Discussion

Heme release has been proposed to play a role in IRI related kidney injury based on the findings that heme levels and HO-1 expression in the microsome (heme-rich) fraction of kidneys are increased after IRI (25). However, due to lack of a suitable method it was not possible to distinguish between bound-heme (i.e., heme in hemoproteins) and un-bound or loosely bound heme (i.e., labile heme). In particular, the latter fraction of heme has been proposed to play a detrimental role in pathophysiological conditions (26). In the current study, we extend these earlier findings and report that IRI causes an increase of the labile heme fraction in renal tissue. Importantly, the levels of labile heme correlated with the duration of warm ischemia time and with the severity of inflammation following IRI. Furthermore, treatment with the heme-scavenger albumin reduced the expression of IRI-related inflammatory markers.

### Source of Labile Heme in the Kidney Following IRI

Kidney after heart has the highest mitochondrial abundance (27) and it is feasible that IRI alters mitochondrial heme synthesis to increase labile heme levels. In support of this notion are the findings that failing human hearts have increased levels of heme accompanied by increased expression of the heme synthesizing enzyme 5′-aminolevulinate synthase 2 (ALAS2) (28). Moreover, increased heme synthesis led to exacerbated injury after coronary ligation in ALAS2 transgenic mice (29). It remains to be evaluated if ischemic injury also alters heme synthesis in the kidney. Independently, an increase in labile heme levels can also be a result of heme release from unstable hemoproteins such as cytochromes due to mitochondrial damage. Corroborating this notion are the findings that inhibitors of cytochrome P450 prevent cell injury by attenuating (heme-derived) iron release and hydroxyl radical formation after reoxygenation of kidney tubular epithelial cells *in vitro* (30). Alternatively, local hemolysis that occurs during IRI or myoglobin released during tissue injury could also contribute to the pool of extracellular labile heme. We have previously shown that prolonged IRI caused substantial impairment of renal perfusion (31) which may result in trapping and degradation of erythrocytes.

### Underlying Mechanisms of Heme-Mediated Inflammation After IRI

The findings of this study indicate that the increase in labile heme levels correlates with the degree of inflammation after prolonged ischemia time. Heme can promote and aggravate inflammation by diverse mechanisms. In endothelial cells, heme induces the production of various adhesion molecules, including E-selectin, P-selectin, intercellular adhesion molecule 1 (ICAM-1), and vascular cell adhesion molecule 1 (VCAM-1) (32, 33), thus promoting leukocyte infiltration. In accordance with this notion, neutrophil infiltration was substantially increased after prolonged ischemia time. Additionally, heme has also been implicated in the secretion of the neutrophil chemoattractant CXCL2 (34). Neutrophil infiltration was mainly observed in the outer medulla, the area with the lowest oxygen tension and consecutively with the highest extent of hypoxia (24).

Our findings also indicate that renal expression of the anaphylatoxin receptors (C5aR1, C5aR2, and C3aR) is induced by prolonged ischemia. Both C3a and C5a are known to promote IRI (35) and C3aR and C5aR deficient mice are protected from IRI-induced damage (35, 36). As a mediator of complement activation, heme has gained increasing appreciation in recent years. Intravascular hemolysis has been shown to activate the complement system via the alternative pathway, which in turn has been implicated in the pathogenesis of atypical hemolytic uremic syndrome (aHUS) (37, 38). More recently, heme-mediated complement activation via the alternative pathway has been shown to be involved in the pro-inflammatory activation of leukocytes (39). Noteworthy, it has been reported that kidney biopsies from sickle cell disease nephropathy patients showed deposits of C3 and C5, which was also observed in a mouse model of sickle cell disease (38). It may well be that the increased expression of C5aR1, C5aR2, and C3aR observed in this study are secondary effects of heme-mediated complement activation. Further studies are required to elucidate the interplay of labile heme and anaphylatoxin receptors by using genetically modified mouse models in renal IRI and kidney transplantation (36, 40). Heme has also been proposed to be a danger associated molecular pattern (DAMP) (41) that can amplify TLR4 signaling. Accordingly, the *in vitro* findings of the current study show that the LPS-induced expression of the pro-inflammatory cytokine IL-6 is aggravated in the presence of heme in bone-marrow derived macrophages which is in accordance with a previous report in peritoneal macrophages after LPS stimulation (21).

### Scavenging Labile Heme as a Therapeutic Strategy Against IRI-Mediated Kidney Injury

In this study treatment with the heme scavenger human serum albumin (HSA) prior to IRI reduced pro-inflammatory cytokine release and C3aR expression in the renal tissue after prolonged IRI. HSA is broadly used in the clinic for the treatment of patients with ascites and chronic liver disease (42, 43) and in the context of plasmapheresis in certain renal diseases (44). However, it is noteworthy that the serum protein hemopexin has a markedly higher binding affinity for heme than albumin. While hemopexin has been shown to counteract heme-mediated complement activation and pro-inflammatory responses in mouse models it has not been approved for therapeutic applications in clinical practice (45, 46). It might well be that preventive strategies using hemopexin could be more beneficial than albumin and future comparative studies are required to understand if heme scavengers can prevent renal IRI-mediated inflammation or delayed graft function after kidney transplantation. Due to the local malperfusion after IRI elaborated strategies to deliver heme scavengers to the kidney might be necessary. In the context of transplantation normothermic machine perfusion strategies (47) might prevent the release of labile heme in allografts. Additionally, the method can also be used for drug delivery to allografts prior to implantation (48). Introducing heme scavengers at the time of *ex vivo* perfusion either directly or in a targeted fashion using drug-delivery systems such as liposomes (49) might also be a viable therapeutic strategy to counter labile heme.

### Limitations

Our study focuses on the early time points after IRI and has only a short follow-up of 2 h after albumin treatment. Further experiments are needed to investigate heme-binding strategies in the context of IRI and to investigate long term effects on renal function such as progressive inflammation and renal fibrosis.

In conclusion, our results indicate that prolonged ischemia time after IRI enhances labile heme levels in renal tissue, which contribute to complement activation, inflammation and AKI. Future studies are warranted to understand if scavenging labile heme can attenuate AKI and delayed graft function after kidney transplantation. Developing clinical applicable therapeutic strategies to reduce the burden of labile heme in the ischemic organ might result in improved long term outcome after solid organ transplantation.

## Data Availability Statement

All datasets generated for this study are included in the article/supplementary material.

## Ethics Statement

The animal study was reviewed and approved by Lower Saxony State department for animal welfare and food protection.

## Author Contributions

FG: designed, supervised all aspects of the study and drafted the manuscript. LW, NS, VV, RC, SR, KM, AT, PP, RG, M-SJ, and FG: experimental conduct. FG, LW, VV, and SI: discussion and wrote the manuscript. RC and SR: animal surgeries. IT, JB, NM, KD, MH-L, CK, RL, and HH: discussion of results. All authors participated in the interpretation of data, editing, and approval of the manuscript.

### Conflict of Interest

The authors declare that the research was conducted in the absence of any commercial or financial relationships that could be construed as a potential conflict of interest.
